# Investigation of the Physiology of the Obligate Alkaliphilic *Bacillus marmarensis* GMBE 72^T^ Considering Its Alkaline Adaptation Mechanism for Poly(3-hydroxybutyrate) Synthesis

**DOI:** 10.3390/microorganisms9020462

**Published:** 2021-02-23

**Authors:** Yağmur Atakav, Orkun Pinar, Dilek Kazan

**Affiliations:** 1 Department of Bioengineering, Faculty of Engineering, Marmara University, Kadıkoy-İstanbul 34722, Turkey; yatakav@atu.edu.tr; 2 Department of Bioengineering, Faculty of Engineering, Adana Alparslan Türkeş Science and Technology University, Sarıcam-Adana 01250, Turkey

**Keywords:** *Bacillus marmarensis*, polyhydroxybutyrate (PHB), lactose, alkaline adaptation

## Abstract

The novel extreme obligate alkaliphilic *Bacillus marmarensis* DSM 21297 is known to produce polyhydroxybutyrate (PHB). However, the detailed mechanism of PHB synthesis in *B. marmarensis* is still unknown. Here, we investigated which metabolic pathways and metabolic enzymes are responsible for PHB synthesis in order to understand the regulatory pathway and optimize PHB synthesis in *B. marmarensis*. In accordance with the fact that beta-galactosidase, 3-hydroxyacyl-CoA dehydrogenase, and Enoyl-CoA hydratase together with acyl-CoA dehydrogenase and lipase were annotated in *B. marmarensis* according to the RAST server, we used glucose, lactose, and olive oil to understand the preferred metabolic pathway for the PHB synthesis. It was found that *B. marmarensis* produces PHB from glucose, lactose, and olive oil. However, the highest PHB titer and the highest amount of PHB synthesized per dry cell mass (Y_P/X_) were achieved in the presence of lactose, as compared to glucose and olive oil. Additionally, in the absence of peptone, the amount of PHB synthesized is reduced for each carbon source. Interestingly, none of the carbon sources studied yielded an efficient PHB synthesis, and supplementation of the medium with potassium ions did not enhance PHB synthesis. According to these experimental results and the presence of annotated metabolic enzymes based on the RAST server, PHB accumulation in the cells of *B. marmarensis* could be improved by the level of the expression of 3-hydroxybutyryl-CoA dehydrogenase (1.1.1.157), which increases the production of NADPH. Additionally, the accumulation of 3-hydroxyacyl-CoA could enhance the production of PHB in *B. marmarensis* in the presence of fatty acids. To our knowledge, this is the first report investigating the regulatory system involved in the control of PHB metabolism of *B. marmarensis*.

## 1. Introduction

Nowadays, manufacturing, consumption, and improper disposal rates of petroleum-based plastics are harming the environment due to their non-biodegradable characteristics. Biopolymers have become significant alternatives to traditional petroleum-based plastics with their eco-friendly properties [1,2]. Within this framework, several attempts have been made to produce biopolymers from renewable sources to cope with environmental pollution. Among the different types of biopolymers, polymers from a microbial origin, such as polyhydroxyalkanoates (PHA) and exopolysaccharides, have become very important, because their characteristics retain the desirable properties of synthetic polymers and they are used in a wide variety of applications [3].

Among different types of microbial polymers, poly(3-hydroxybutyrate) (PHB) is one of the most attractive and well-studied biopolymers belonging to the polyhydroxyalkanoates (PHA) family [1,4,5,6]. PHB is now a promising candidate for packaging, medicinal, agricultural, and automotive applications because of its non-toxic, biocompatible, and biodegradable properties [6,7,8,9]. However, its high production cost, fragility, rigidity, and thermally unstable characteristics during processing impede the development of the PHB market [10]. Therefore, research activities are continued in order to produce PHB with desired properties using cost-effective processes. PHB can be synthesized by several microorganisms as an energy storage compound under stress conditions due to the limitation of different essential nutrients [1,5,6]. Although various Gram-negative bacteria such as *Burkholderia sacchari*, *Alcaligenes eutrophus* (*Cupriavidus necator*), and *Alcaligenes latus* (*Azohydromonas australica*) are well-known microorganisms for PHB production, Gram-positive bacteria such as *Bacillus megaterium* are also encouraging in terms of PHB production due to the lack of endotoxin synthesis [11,12,13].

Recently, *Bacillus marmarensis*, an extreme obligate alkaliphilic and protease-producing bacterium, has been reported as a sustainable microorganism for biorefining, allowing sterilization to be eliminated without the risk of contamination. Additionally, the metabolic pathway for poly(3-hydroxybutyrate) was annotated [14,15,16]. Within the frame of references, the production of PHB from *B. marmarensis* was evaluated for the production of poly(3-hydroxybutyrate), and the effect of environmental conditions and medium components on PHB synthesis was investigated [17]. The PHB obtained from *B. marmarensis* is more thermostable compared to the commercial one and can be used for the composite production for nanofiber scaffold manufacturing [17]. Moreover, the obligate alkaliphilic *B. marmarensis* DSM 21297 is a promising candidate for industrial bioprocesses and PHB production. In our previous work, it was observed that PHB synthesis was initiated after the decrease in pH of the cultivation medium below pH 9.0, although *B. marmarensis* is an obligate alkaliphilic bacteria and can grow in extreme alkaline conditions [17]. The decrease in pH values of the medium during the cultivation of *B. marmarensis* was explained by the alkaline adaptation of *B. marmarensis* with H^+^ secretion due to the presence of additional enzymes [15]. To understand the unique adaptation mechanism of *B. marmarensis* in detail, our group also studied the alkaliphilic adaptation strategy of *B. marmarensis* using a multi-omics approach that included transcriptomics and proteomics [18]. According to our results, the cell membrane of *B. marmarensis* is altered by changing the peptidoglycan constituents together with peptide moieties due to the remodeling of the peptidoglycan layer. Because of these changes in the cell membrane, lipid mobility is decreased and H^+^ secretion is also altered to establish intracellular pH. As was indicated by Wernick et al. [15], potassium ions are responsible for alkaline adaptation in strain DSM 21297 [18].

When the effect of glucose, sucrose, fructose, and maltose on PHB synthesis in *B. marmarensis* was investigated, interestingly, it was determined that the highest titer of PHB was achieved in the absence of carbon sources (sucrose, maltose, fructose, and glucose), and the synthesis of PHB in *B. marmarensis* was improved by the addition of inorganic salts such as MgSO_4_.7H_2_O and KH_2_PO_4_ to the medium containing 1% glucose. The induction of PHB synthesis by mineral salts was explained by a unique adaptation mechanism of *B. marmarensis* and an uncommon KtrB, the K^+^ uptake protein present in *B. marmarensis* cells [15]. However, it is a well-known fact that PHB is produced by various bacterial strains via its metabolic pathway based on the conversion of various carbon sources such as glucose, glycerol, sucrose, lactose, maltose, and fructose [19,20,21,22].

Therefore, our surprising result prompted us to investigate the physiology of strain DSM21297 to understand how *B. marmarensis* produces PHB and which metabolic carbon fluxes are dominated for PHB synthesis. Since the highest PHB synthesis was achieved without carbon sources and was increased by the addition of potassium ions together with magnesium ions and glucose, in the present work, the relationship between the unique adaptation mechanism and poly(3-hydroxybutyrate) synthesis of *B. marmarensis* was also evaluated. We hypothesized that *B. marmarensis* might prefer different metabolic carbon fluxes to produce PHB.

To our knowledge, this is the first report to evaluate the physiology of *B. marmarensis* and to understand the relationship between the adaptation mechanism and poly(3-hydroxybutyrate) synthesis in *B. marmarensis.*

## 2. Materials and Methods

### 2.1. Materials

*B. marmarensis* GMBE 72^T^ (DSM 21297) was obtained from DSMZ, German Collection of Microorganisms and Cell Cultures (Braunschweig, Germany). All chemicals used in experiments were purchased from Merck KGaA (Darmstadt, Germany).

### 2.2. Cultivation of B. marmarensis in the Absence of Carbon Sources

A solution containing 5 g/L peptone, 3 g/L meat extract, 10 g/L NaCl, 0.04 g/L Mg_2_SO_4_.7H_2_O, and 0.04 g/L KH_2_PO_4_ was used as carbon-free medium and sterilized in the autoclave at 121 °C for 15 min. The pH of each working medium was adjusted to 8.8 (±0.2), as it was shown to give the highest amount of PHB in our previous work [17], by using a 10% *v*/*v* Na-sesquicarbonate buffer solution in 1000 mL Erlenmeyer flasks with a final volume of 20% of the flask. The sterilization of the buffer solution was achieved via a 0.22 µm cellulose acetate filter. Each flask was inoculated with a 12-h-old preculture by a 1% inoculum size, and cultured at 30 °C at 180 rpm for 24 h [17]. Cells were harvested by centrifuging at 4000× *g* for 20 min, and these cell pellets were used for PHB extraction.

### 2.3. Investigation of the Physiology of B. marmarensis Using K^+^ and M^2+^ Ions and Different Carbon Sources

#### 2.3.1. Effect of K^+^ and Mg^2+^ Ions on PHB Synthesis

In our previous work, we showed that the use of Mg_2_SO_4_.7H_2_O and KH_2_PO_4_ enhances PHB production and that the potassium transport system is responsible for the alkaline adaptation of *B. marmarensis* [17,18]. Therefore, to investigate the effect of Mg^2+^ and K^+^ ions on PHB synthesis, the carbon-free medium was supplemented with 0.02%, 0.04%, 0.08%, and 0.16% (*w*/*v*) of Mg_2_SO_4_.7H_2_O and KH_2_PO_4_ along with 1% (*w*/*v*) glucose. After incubation of *B. marmarensis* at 30 °C and 180 rpm for 24 h, cells were harvested by centrifugation at 4000× *g* for 20 min, and these cell pellets were used for PHB extraction.

#### 2.3.2. Effect of Lactose and Galactose on PHB Synthesis

Since we determined the beta-galactosidase (EC 3.2.1.23) based on the *B. marmarensis* genome annotation using the RAST (Rapid Annotations using Subsystems Technology) server [23], the effect of lactose on PHB synthesis was investigated and compared with glucose to predict the physiology of *B. marmarensis* and to understand how this strain synthesizes PHB. The carbon-free medium including 1% (*w*/*v*) glucose was used as the control medium, and glucose was replaced by lactose at a concentration of 1% (*w*/*v*) to analyze the PHB synthesis from lactose. In addition, the production of PHB in *B. marmarensis* was evaluated during growth in the presence of galactose in comparison with glucose, since galactose is the product of lactose hydrolysis. The two used concentrations of each of these two alternative carbohydrates were 1% and 10% (*w*/*v*). Cultivation was carried out for 24 h and 48 h. After cultivation at 30 °C and 180 rpm for 24 h, cells were harvested by centrifuging at 4000× *g* for 20 min, and these cell pellets were used for PHB extraction.

#### 2.3.3. Effect of Different Nitrogen Sources and Nitrogen Limitation on PHB Synthesis

To investigate the effect of various nitrogen sources on the efficiency of PHB production, nitrogen was introduced into a carbon-free medium as a combination of both organic and inorganic nitrogen sources so that the total nitrogen concentration remained constant (0.8%, *w*/*v*). Yeast extract, beef extract, meat extract, malt extract, and tryptone were used as organic sources; ammonium chloride, ammonium nitrate, ammonium sulfate, and sodium nitrate were used as inorganic sources. A carbon-free medium including 0.5% peptone and 0.3% meat extract was used as control. Each flask was inoculated with a 12-h-old preculture by a 1% inoculum size and cultured at 30 °C at 180 rpm for 24 h [17]. Cells were harvested by centrifuging at 4000× *g* for 20 min, and these cell pellets were used for PHB extraction.

Since C/N ratio is very important for PHB expression, a nitrogen-limited medium including (NH_4_)_2_SO_4_ (2 g/L), KH_2_PO_4_ (2 g/L), MgSO_4_.7H_2_O (0.2 g/L), Na_2_HPO_4_ (0.6 g/L), and yeast extract (0.2 g/L) was used [24] and was supplemented with glucose at a 1% (*w*/*v*) concentration. Moreover, the effect of peptone removal from the medium was also evaluated since it has been shown that the removal of peptone from the medium induces lactate and succinate synthesis [16]. Therefore, peptone and meat extracts in the carbon-free medium were replaced by yeast extract at a concentration of 0.8 and 1.6 g/L. After cultivation in each medium at 30 °C and 180 rpm for 24 h, cells were harvested by centrifuging at 4000× *g* for 20 min, and these cell pellets were used for PHB extraction.

#### 2.3.4. Effect of Vegetable Oil as Carbon Source on PHB Synthesis

When the genome of *B. marmarensis* was annotated using the RAST server, lipase enzyme was determined. Hence, we evaluated the synthesis of PHB from vegetable oil such as olive oil. The carbon-free medium was modified by replacing peptone and meat extracts with the same amount of yeast extract and 1% olive oil (*v*/*v*). The cultivation of *B. marmarensis* was achieved at 30 °C and 180 rpm for 24 h, cells were harvested by centrifuging at 4000× *g* for 20 min, and these cell pellets were used for PHB extraction.

Additionally, *B. marmarensis* was cultivated in a carbon-free medium supplemented with 1% (*w*/*v*) concentrations of glucose, lactose, and olive oil. The PHB titer and Y_P/X_ were also evaluated and compared to understand the primary substrate for PHB synthesis.

In all experiments conducted for the cultivation of strain DSM 21297, its growth was estimated by measuring the optical density at 600 nm, and the cell dry mass (CDM) was calculated using the relationship between OD_600_ and CDM (g/L).

### 2.4. Extraction of PHB from B. marmarensis

To extract crude PHB from *B. marmarensis* cells, for an effective cell disruption, wet cells were treated with sodium hypochlorite (6–14%) (1:10, g wet cells/mL sodium hypochlorite) for 1 h in a rotary shaker at 300 rpm and 37 °C. Afterwards, PHB was extracted from the cell suspension by using chloroform as an extraction solvent. The same volume of chloroform with hypochlorite (1:1, *v*/*v*) was added onto the hypochlorite mixture and incubated at 60 °C for 2 h to dissolve PHB in the chloroform phase. The chloroform phase was then separated in a separating funnel and mixed with ice-cold solvent mixture (1:1 *v*/*v*, ethanol/acetone) to precipitate PHB [25]. This precipitate was then dried to determine the amount of PHB produced from strain DSM 21297.

### 2.5. Determination of Reducing Sugar Concentration

The amount of reducing sugars in the culture media was determined by a modified version of the DNS method described by Miller et al. [26]. The standard calibrating curves prepared by using glucose and galactose were used to determine the amount of reducing sugars consumed.

## 3. Results

### 3.1. The Effect of K^+^ and Mg^2+^ Ions on PHB Synthesis

It was reported that the K^+^ uptake protein was responsible for the survival of *B. marmarensis* under alkaline conditions, and the DSM 21297 strain adapted to alkaline pH by lowering the pH of the medium due to the formation of H^+^ ions [15]. Therefore, in our previous work, PHB synthesis was improved by 0.04% MgSO_4_.7H_2_O and KH_2_PO_4_ addition in the presence of glucose, and the pH of the medium dropped to a level below 9.0 during the cultivation [17].

**Figure 1:**
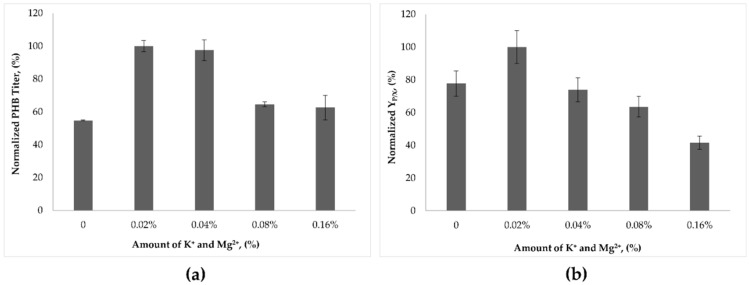
Effect of K^+^ and Mg^2+^ ions on PHB synthesis. (**a**) Normalized amount of PHB titer (%). (**b**) Normalized Y_P/X_ (%).

In the present work, the effect of different concentrations of K^+^ ions together with Mg^2+^ ions on PHB synthesis in *B. marmarensis* was investigated to evaluate the relationship between PHB synthesis and alkaline adaptation. The normalized titer of PHB (%) and product yield based on dry cell biomass (Y_P/X_) at different salt concentrations are given in Figure 1a,b.

As shown in Figure 1a, the highest PHB titer was obtained when the total content of MgSO_4_.7H_2_O and KH_2_PO_4_ reached 0.02% and 0.04%. Removal of mineral salts or an increase in both MgSO_4_.7H_2_O and KH_2_PO_4_ concentrations by four and eight times led to a decrease in the PHB titer by 40%. To analyze the behavior of *B. marmarensis* together with PHB synthesis, Y_P/X_ values were evaluated at different concentrations of mineral salts (Figure 1b). The addition of 0.02% MgSO_4_.7H_2_O and KH_2_PO_4_ enhanced PHB synthesis. However, increasing salt concentrations above 0.02% resulted in a decrease in Y_P/X_.

### 3.2. Effect of Lactose, Galactose, and Abundant Carbon Sources on PHB Synthesis

**Figure 2:**
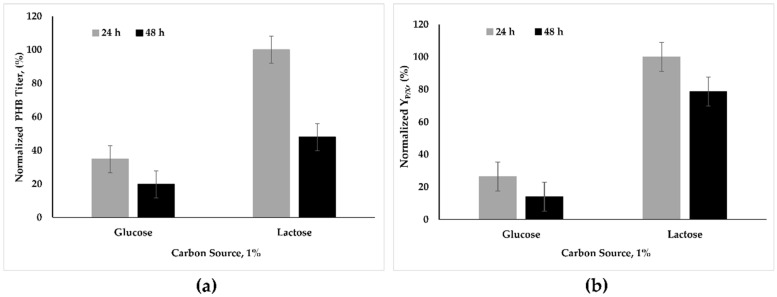
Effect of lactose on PHB synthesis. (**a**) Normalized amount of PHB titer (%). (**b**) Normalized Y_P/X_ (%).

According to the RAST server, a tool for annotating prokaryotic genomes [23], the *B. marmarensis* genome contains beta-galactosidase (EC 3.2.1.23), an enzyme that utilizes lactose. Therefore, the production of PHB from lactose in comparison with glucose was evaluated in view of the high availability of dairy waste, including lactose (Figure 2).

In the presence of 1% glucose and lactose at pH 8.8 (±0.2) and 30 °C, the amounts of both PHB and Y_P/X_ obtained for glucose were lower than for lactose. For 24 h of cultivation, the amount of PHB obtained with glucose was almost 65% lower than that obtained with lactose. A prolonged cultivation time (48 h) caused a moderate decrease in the amount of PHB obtained in the presence of glucose; however, in the presence of lactose, the amount of PHB was halved (Figure 2a). As for the yield of PHB (Y_P/X_), it was almost 75% lower for glucose compared to lactose (Figure 2b). A prolonged cultivation time (48 h) caused a dramatic decrease in the PHB yield for glucose, but for lactose, the decrease of PHB yield was almost 20% (Figure 2b).

**Figure 3:**
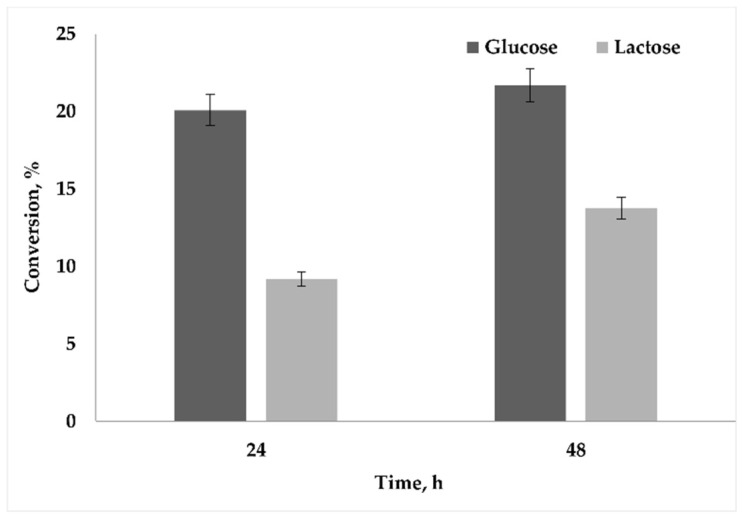
Consumption of glucose and lactose during the cultivation period.

Comparing the glucose and lactose consumption, during the first 24 h (Figure 3), 20% and 10% of the initial glucose and lactose concentrations, respectively, were consumed. However, during the prolonged cultivation period (48 h), a slight increase was observed for glucose consumption (21% of the initial glucose concentration). As for lactose, the conversion was increased from 9.16% to 13.75%. According to these results, we assumed that lactose rather than glucose induces the production of PHB in *B. marmarensis*.

**Figure 4:**
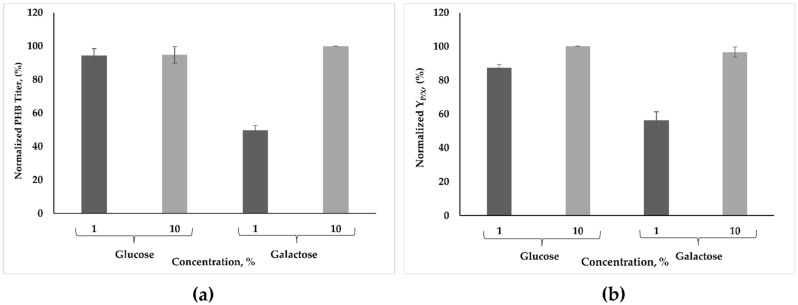
Effect of galactose on PHB synthesis. (**a**) Normalized amount of PHB titer (%). (**b**) Normalized Y_P/X_ (%).

To ascertain whether the cells of *B. marmarensis* utilize galactose to produce poly(3-hydroxybutyrate), PHB titer and Y_P/X_ were determined in the presence of galactose at 1% and 10% concentrations and compared with glucose (Figure 4). It is evident from the data of Figure 4 that *B. marmarensis* utilized galactose for the production of PHB; however, the PHB titer and Y_P/X_ index obtained with 1% galactose were almost half that obtained with 1% glucose after 24 h of incubation. A further tenfold increase in the concentration of galactose led to a significant increase in the production of PHB, and both the PHB titer and Y_P/X_ index reached the values obtained with glucose at concentrations of 1% and 10%. Interestingly, a tenfold increase in glucose concentration did not affect the PHB titer or Y_P/X_ index.

The conversion of galactose to glucose is achieved by the conversion of galactose to UDP-galactose. UDP-galactose 4-epimerase (EC 5.1.3.2), which is one of the enzymes responsible for this catalysis in the Leloir pathway [27], was annotated in *B. marmarensis* according to the RAST server that indicates the galactose consumption by the cells of *B. marmarensis*.

### 3.3. Effect of Nitrogen Sources and Nitrogen Limitation on PHB Synthesis

**Figure 5:**
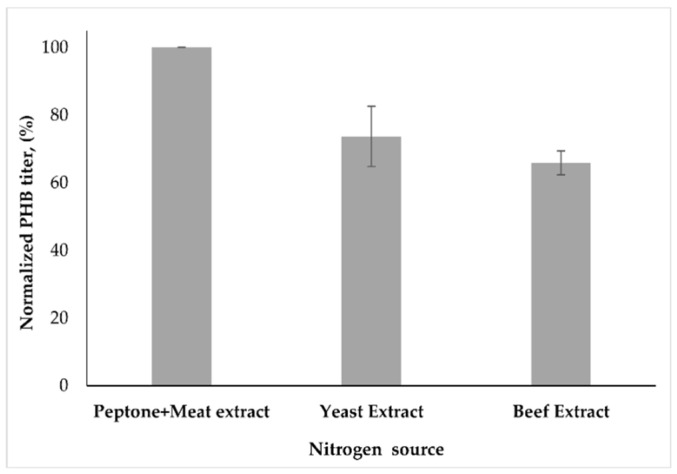
Effect of nitrogen sources on PHB synthesis.

To understand the relative contribution of nitrogen sources in PHB production, yeast extract, beef extract, meat extract, malt extract, and tryptone were replaced with the nitrogen sources (peptone and meat extract) in a carbon-free medium, and PHB titer was determined in *B. marmarensis*. Since *B. marmarensis* did not efficiently grow in the presence of meat extract, malt extract, and tryptone, the effect of yeast extract and beef extract were evaluated for PHB production in *B. marmarensis* (Figure 5). It is evident from Figure 5 that the PHB production in *B. marmarensis* attained its maximum level at 5 g/L peptone and 3 g/L meat extract after 24 h of incubation. Replacing peptone and meat extract with yeast extract and beef extract resulted in approximately 25% and 35% decreases in PHB titer, respectively. On the other hand, changing the yeast extract concentration from 0.8% to 1.6% increased the PHB titer, while the Y_P/X_ remained constant. Moreover, in the case of inorganic nitrogen sources (i.e. a nitrogen-limiting medium supplemented with 1% glucose), the cells of *B. marmarensis* did not show any growth.

### 3.4. Effect of Vegetable Oil and Peptone Removal on PHB Synthesis

To investigate the contribution of vegetable oil in PHB production in *B. marmarensis*, PHB content was measured in the cells of *B. marmarensis* grown in the presence of 1% olive oil, and for comparison, in the presence of glucose and lactose (Table 1). Since the production of the highest amount of lactate and succinate in the absence of peptone in *B. marmarensis* cells is reported [16], we hypothesized that *B. marmarensis* might shift the carbon flux route from the TCA cycle to PHB synthesis in the presence of peptone. Instead of using meat extract, yeast extract was used as the nitrogen source in the absence of peptone considering that the growth of *B. marmarensis* was shown to be low in the carbon-free medium supplemented with meat extract.

The removal of peptone and the use of yeast extract as the nitrogen source caused a decrease in both the PHB titer and Y_P/X_ in the three carbon sources evaluated. The presence of yeast extract reduced the PHB titer from 140 ± 32.20 mg/L to 20 ± 4.860 mg/L in lactose, from 60 ± 16.60 mg/L to 35 ± 6.650 mg/L in glucose, and from 30 ± 4.210 mg/L to 10 ± 2.160 mg/L in olive oil, while Y_P/X_ values were decreased from 125 ± 25.40 mg PHB/g dry cell mass to 30.30 ± 3.636 mg PHB/g dry cell mass in lactose and from 25 ± 3.762 mg PHB/g dry cell mass to 12.50 ± 2.625 mg PHB/g dry cell mass in olive oil. For glucose, the yield of PHB obtained from the yeast extract was very close to the Y_P/X_ obtained in the presence of meat extract and peptone. The lowest PHB titer (10 ± 2.160 mg/L) and Y_P/X_ (12.50 ± 2.625 mg PHB/g dry cell mass) were obtained in olive oil in the presence of yeast extract as the nitrogen source.

**Figure 6:**
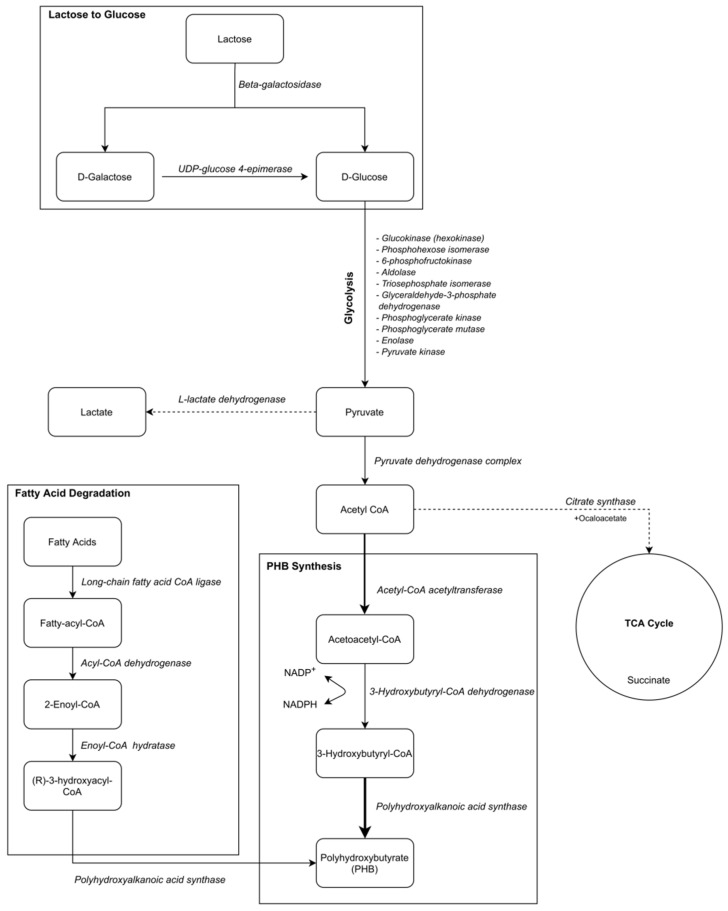
Scheme for PHB synthesis pathway including responsible enzymes based on RAST server annotations of *B. marmarensis* genome (continuous line arrows: paths for PHB synthesis; dotted line arrows: paths that cause decrease in PHB synthesis).

Although lactose-induced PHB synthesis and the PHB titer obtained from lactose were almost two times higher than that obtained from glucose, the amount of PHB is still lower than that of currently known industrial microorganisms. The lower PHB synthesis and decrease in PHB titer at a prolonged incubation period prompted us to investigate the annotation of enzymes involved in PHB degradation. According to annotations on the RAST server, acyl-CoA dehydrogenase (EC 1.3.8.7) and enoyl-CoA hydratase (EC 4.2.1.17)/3-hydroxyacyl-CoA dehydrogenase (EC 1.1.1.35) were determined. It was reported that an enoyl-CoA is produced by the action of an acyl-CoA dehydrogenase, and an enoyl-CoA is then converted to (*S*)–3-hydroxyacyl–CoA stereoselectively by the catalysis of an enoyl-CoA hydratase. After that, a 3-hydroxyacyl–CoA dehydrogenase catalyzes the reaction to convert (*S*)–3-hydroxyacyl–CoA to 3-ketoacyl–CoA, which is cleaved by a 3-ketoacyl–CoA thiolase to form acetyl-CoA [28]. Although acyl-CoA dehydrogenase (EC 1.3.8.7) and enoyl-CoA hydratase (EC 4.2.1.17)/3-hydroxyacyl-CoA dehydrogenase (EC 1.1.1.35) catalyze reactions of PHB degradation, the enzyme that catalyzes depolymerization of PHB to 3-hydroxybutryl-CoA (PHB depolymerase) was not annotated. To explain the relationship between the annotated enzymes and target substrates that might play a role in the production of poly(3-hydroxybutyrate) in *B. marmarensis*, the annotated *B. marmarensis* enzymes and target substrates are tabulated in Table 2, and the relationship is schematically presented in Figure 6.

## 4. Discussion

The results presented here show that the addition of K^+^ ions together with Mg^2+^ ions at a concentration of 0.02% and 0.04% enhanced PHB synthesis. However, raising K^+^ ions together with Mg^2+^ ions above 0.02% reduced Y_P/X_ (Figure 1a,b). The potassium ion uptake system, including the unique KtrB K^+^ uptake protein, is responsible for alkaline survival of *B. marmarensis.* The strain DSM 21297 adapted to alkaline pH by lowering the medium pH due to the formation of H^+^ ions. However, K^+^ ions, together with Mg^2+^ ions, are not directly responsible for the use of glucose for the production of PHB, since an increase in the content of potassium ions in the presence of glucose does not increase the PHB titer. The findings on the effect of K^+^ ions together with Mg^2+^ ions on PHB did not provide direct confirmation for the requirement of K^+^ ions for PHB synthesis. Potassium-dependent transporters, the mutation in AtpC, H^+^ secretion due to the presence of an additional enzyme, and the unique KtrB protein are responsible for maintaining the cytoplasmic pH below external pH [16,17,18]. However, these mechanisms are not directly responsible for PHB synthesis. This suggests that DSM 21297 first adapted to extreme pH values through its unique adaptation mechanisms and then began to grow and synthesize PHB.

The obligate alkaliphilic strain DSM 21297 is a promising candidate for industrial bioprocesses and has the ability to produce PHB. However, its ability to produce PHB is too low and needs to be increased for further application of the strain [17]. To increase productivity, one of the most effective approaches is to identify metabolic pathways using the complete genome sequences of microorganisms and develop a metabolic network to design cost-effective manufacturing processes [29]. Additionally, it is very important to know the regulatory circuits that control PHB metabolism [30]. The production of PHB generally increased under stress conditions in the presence of carbon sources, but interestingly, *B. marmarensis* produced PHB in the absence of carbon sources, and PHB titer was reduced by the addition of glucose [17]. Therefore, in the present work, the physiological response of the strain DSM 21297 was evaluated to understand how *B. marmarensis* DSM 21297 produces PHB, and to determine which metabolic carbon fluxes are responsible for PHB production.

Low consumption and a slight increase in the utilization of glucose (Figure 3) might be explained with sugar uptake mechanisms of *B. marmarensis*. In bacteria, the uptake of essential nutrients and the efflux of the toxic compounds are achieved by the major facilitator superfamily (MFS) transporter, while ATP-binding cassette (ABC) transporters are responsible for efflux and the uptake of metabolites, vitamins, amino acids, lipids, peptides, ions, and drugs [31]. It was determined in our previous proteomic and transcriptomic analysis of *B. marmarensis* that most of the ATP transporters and MFS transporters except one were downregulated at extreme pH [18]. This result directed us to annotate the presence of the phosphoenolpyruvate (PEP)-dependent phosphotransferase system (PTS), which is responsible for the uptake of carbohydrates, mainly hexoses, hexitols, and disaccharides [32]. In the present work, several PEP-PTS systems (including fructose-specific IIA, IIB, and IIC components; glucose-specific IIA, IIB, and IIC components; maltose and glucose-specific IIB and IIC components; sucrose-specific IIB and IIC components; mannitol-specific IIA, IIB, and IIC components; and galactitol-specific IIA, IIB, and IIC components) were determined by genome annotation using the RAST system. Additionally, beta-galactosidase (EC 3.2.1.23) was also annotated. Previously, we also assessed sucrose, maltose, and fructose as carbon sources for PHB production [17]. Although the amounts of PHB obtained from sucrose, fructose, and maltose were slightly higher compared to glucose, the values were lower than those obtained without sugar supplementation [17]. These results led us to the use of lactose as a carbon source for PHB production in *B. marmarensis*, since *Bacillus* species have been shown to produce PHB from lactose-supplemented media [33]. To produce PHB from lactose, the first step of biosynthesis is the enzymatic hydrolysis of lactose to glucose and galactose. Since beta-galactosidase hydrolyzes lactose to glucose and galactose, lactose was tested for PHB production (Figure 2a,b) due to the annotation of beta-galactosidase in *B. marmarensis* genome using the RAST server.

Our results showed that PHB titer (mg/L) and Y_P/X_ increase in the presence of lactose (Figure 2a,b), and the lactose consumption increases at a prolonged incubation time (48 h) (Figure 3). Generally, bacteria have the ability to utilize different types of carbon sources. The specific substrates induce the expression of genes that are responsible for the catabolic enzymes and repressed by carbon sources and glucose. Both induction and repression processes are accommodated by the PTS system, which is responsible for the transportation and phosphorylation of sugars. *Bacillus subtilis*, one of the model organisms of *Bacillus* species, uses glucose as carbon and energy sources. In *B. subtilis*, glucose is transported by a PTS system encoded by *ptsGHI* operon. Glucose induces the expression of this operon, and the induction requires the GlcT anti-terminator protein. In the absence of glucose, the activity of GlcT is negatively controlled by glucose permease [34].

The annotation of the GlcT and beta-glucoside *bgl* operon anti-terminator, the BglG family, and UDP-glucose 4-epimerase showed that *B. marmarensis* utilizes glucose and galactose as carbon sources for its energy, survival, and PHB synthesis. Although glucose did not induce PHB production, the increase in galactose concentration induced PHB synthesis in *B. marmarensis*. On the other hand, the PHB synthesis was stimulated by lactose. Bacteria synthesize PHB by the conversion of acetyl CoA to acetoacetyl-CoA and later to β-hydroxybutyryl-CoA (3-hydroxybutyryl-coenzyme A). The concentration of dihydronicotinamide adenine dinucleotide phosphate (NADPH) and a high ratio of NADPH/nicotinamide adenine dinucleotide phosphate (NADP) are crucial to stimulate PHB synthesis [30]. The enhancement of the PHB synthesis by lactose might be due to the production of NADPH at the desired level for the activity of 3-hydroxybutyryl-CoA dehydrogenase (1.1.1.157).

The production of PHB is achieved through the NADPH-dependent pathway by converting acetyl-CoA to PHB [30]. Since succinate and lactate are produced during cultivation [16], *B. marmarensis* prefers either the citric acid cycle (the TCA cycle) or PHB synthesis. This situation could be an indicator of a competition between these two alternative routes in the metabolic pathway. In the presence of lactose, the enzyme responsible for PHB formation could be induced, and *B. marmarensis* prefers to produce more PHB from lactose.

The decreases in PHB titer and Y_P/X_ in the absence of peptone (Table 1) are in agreement with the results reported by Wernick et al. [16]. They noted that using a minimal medium including glucose enhanced the amount of lactate and succinate, while the use of excess glucose and the removal of peptone from the medium resulted in the highest amount of lactate and succinate [16]. The results related to the decrease in the amount of PHB for olive oil, glucose, and lactose in the absence of peptone also might have proved that the removal of peptone from the medium could orient *B. marmarensis* to a TCA cycle for the production of succinate to maintain its metabolic activity. The decrease in PHB production due to the presence of yeast extract in *B. marmarensis* culture supports our hypothesis.

Besides the different types of carbohydrates, it is reported that PHA can be produced by using vegetable oils as carbon sources [35]. Therefore, we evaluated the usage of olive oil as a carbon source for PHB production from *B. marmarensis* to understand the alternative metabolic carbon flux for PHB synthesis, since lipase enzyme was annotated via the RAST server. Additionally, according to the annotation of the genome of *B. marmarensis*, acyl-CoA dehydrogenase (EC 1.3.8.7) and 3-hydroxyacyl-CoA dehydrogenase (1.1.1.35)/Enoyl-CoA hydratase (EC 4.2.1.17) were determined. Long chain fatty acid CoA-ligase is responsible for breaking down long-chain fatty acids into fatty acyl-CoA molecules in the first step of fatty acid metabolism. Fatty acyl-CoA is then converted to 2-enoyl-CoA by acyl-CoA dehydrogenase. Based on the literature, enoyl-CoA hydratase (EC 4.2.1.17) is involved in fatty acid degradation and converts 2-enoyl-CoA to R-3-hydroxyacyl-CoA, an intermediate metabolite in PHB synthesis. R-3-hydroxyacyl-CoA is then converted to PHB by polyhydroxyalkanoic acid synthase (PhaC) [36,37]. Consequently, it was thought that *B. marmarensis* could produce lipase, which could break down triglycerides to fatty acids, and these fatty acids could then be used to produce PHA through the conversion of degradation of fatty acids to acetyl-CoA. Generally, due to the fatty acid beta-oxidation, the acetyl-CoA is increased, and the metabolic flux turned to PHA synthesis [36]. Acetyl-CoA, a branch point for PHA synthesis, is expressed through fatty acid biosynthesis and glycolysis. Velázquez-Sánchez et al. [30] reported that polyhydroxyalkanoic acid synthase (PhaC), which catalyzes the terminal step of the PHA synthesis, is inhibited by the ratio of R-3-hydroxyacyl-CoA/CoA and free CoA. Additionally, the oxidation and the accumulation of 3-hydroxyacyl-CoA are affected by the acetyl CoA/CoA and NADH/NAD ratios that regulate the PHA accumulation. The high ratio of acetyl CoA/CoA and NADH/NAD results in PHA accumulation in the cells [30]. In the case of *B. marmarensis*, efficient PHA accumulation was not found in the cells of *B. marmarensis* growing in the medium including olive oil, which contains a high amount of oleic acid. Although *B. marmarensis* did not accumulate PHB efficiently, a high PHB yield was reported in the literature [38]. The lowest PHB accumulation in *B. marmarensis* cells could be explained by the lowest acetyl CoA/CoA and NADH/NAD ratios. Therefore, 3-hydroxyacyl-CoA might be oxidized, and the NAD level might be increased, which would negatively affect the PHB accumulation. Since branch chain fatty acids are generally enriched in extremophiles [39], it might be preferable to use fatty acids, rather than the accumulation of PHB, for the alkaline adaptation of *B. marmarensis*. There might be a relationship between fatty acid accumulation and lipid mobility that is reduced to adjust intracellular pH [18].

The standard deviations of PHB titer and Y_P/X_ in the presence of glucose, lactose, and olive oil were high (Table 1). These results made us consider that *B. marmarensis* might fluctuate between two metabolic pathways in the presence of carbon sources: energy metabolism and PHB production. Therefore, both the utilization of glucose and galactose as hydrolysis products of lactose and fatty acid utilization require further analysis.

## 5. Conclusions

The objective of this study was to investigate how the novel extreme obligate alkaliphilic *B. marmarensis* DSM 21297 produces PHB and which metabolic routes are responsible for PHB synthesis. Rapid Annotation using Subsystem Technology (RAST) was used to determine enzymes that might play a role in the production of PHB. According to annotation results on the RAST server and the experimental results, the use of glucose, lactose, and olive oil as carbon sources is in agreement with the annotation of the sugar uptake system, the fatty acid degradation pathway, and beta-galactosidase. Comparing the carbon sources studied, lactose is the most promising one for PHB production from *B. marmarensis*. However, understanding the decrease in PHB titer with both glucose and lactose for a prolonged incubation period requires further analysis. In the presence of glucose and olive oil, *B. marmarensis* can shift the PHB synthesis route to the fatty acid biosynthesis route or TCA cycle to maintain its survival. Additionally, the accumulation of PHB does not directly depend on the potassium ion uptake system of strain DSM 21297. Therefore, *B. marmarensis* can synthesize PHB and regulates its metabolism without loss of energy. An increase in the NADPH level and the activity of 3-hydroxybutyryl-CoA dehydrogenase (1.1.1.157) can increase the accumulation of PHB in the cells of *B. marmarensis*. These new findings can be used to improve the novel obligate alkaliphilic *B. marmarensis*. They can broaden the knowledge of PHB production mechanism from obligate alkaliphiles and can potentially be used for further studies to enhance PHB production from *B. marmarensis* and other obligate alkaliphiles.

## Figures and Tables

**Table 1:** Comparison of olive oil, glucose, and lactose by PHB titer (mg/L) and Y_P/X_ (mg PHB/g dry cell mass).

C Source (1%)	N Source	PHB Titer (mg/L)	Y_P/X_ (mg PHB/g Dry Cell Mass)
Lactose	Yeast extract (0.8%)	20 ± 4.86	30.30 ± 3.636
	Meat extract (0.3%) and peptone (0.5%)	140 ± 32.20	125 ± 25.40
Glucose	Yeast extract (0.8%)	35 ± 6.65	32.71 ± 3.108
	Meat extract (0.3%) and peptone (0.5%)	60 ± 16.60	35.29 ± 1.605
Olive oil	Yeast extract (0.8%)	10 ± 2.16	12.50 ± 2.625
	Meat extract (0.3%) and peptone (0.5%)	30 ± 4.21	25 ± 3.762

**Table 2:** The annotated *B. marmarensis* enzymes and their target substrates in the PHB synthesis pathway.

	Enzyme	EC Number	Target Substrate	Product
**Lactose to Glucose and** **Galactose**	Beta-galactosidase	3.2.1.23	Lactose	D-Glucose D-Galactose
**Galactose to Glucose**	UDP-glucose 4-epimerase	5.1.3.2	D-Glucose	D-Galactose
**Glucose to Acetyl-CoA**	Glucokinase (hexokinase)	2.7.1.2	D-glucose	D-glucose 6-phosphate
	Phosphohexose isomerase	5.3.1.9	D-glucose 6-phosphate	D-fructose 6-phosphate
	6-phosphofructokinase	2.7.1.11	D-fructose 6-phosphate	D-fructose 1,6-bisphosphate
	Aldolase	4.1.2.13	D-fructose 1,6-bisphosphate	D-glyceraldehyde 3-phosphate Glycerone phosphate
	Triosephosphate isomerase	5.3.1.1	D-glyceraldehyde 3-phosphate	Glycerone phosphate
	Glyceraldehyde-3-phosphate dehydrogenase	1.2.1.12	D-glyceraldehyde 3-phosphate	3-phospho-D-glyceroyl phosphate
	Phosphoglycerate kinase	2.7.2.3	3-phospho-D-glyceroyl phosphate	3-phospho-D-glycerate
	Phosphoglycerate mutase	5.4.2.1 (5.4.2.11)	3-phospho-D-glycerate	2-phospho-D-glycerate
	Enolase	4.2.1.11	2-phospho-D-glycerate	Phosphoenol pyruvate
	Pyruvate kinase	2.7.1.40	Phosphoenol pyruvate	Pyruvate
	Pyruvate dehydrogenase complex	1.2.4.1 2.3.1.12 1.8.1.4	Pyruvate	Acetyl-CoA
**Acetyl-CoA to PHB Synthesis**	Acetyl-CoA acetyltransferase	2.3.1.9	Acetyl-CoA	Acetoacetyl-CoA
	3-hydroxybutyryl-CoA dehydrogenase	1.1.1.157	Acetoacetyl-CoA	3-Hydroxybutyryl-CoA
	Polyhydroxyalkanoic acid synthase	2.3.1.-	3-Hydroxybutyryl-CoA	Polyhydroxybutyrate (PHB)
**Pyruvate to** **Lactate**	L-lactate dehydrogenase	1.1.1.27	Pyruvate	Lactate
**Acetyl CoA to TCA** **cycle and Succinate**	Citrate synthase	2.3.3.1	Acetyl-CoA + oxaloacetate	Citrate
**Fatty acid** **degradation for PHB** **Synthesis**	Long-chain fatty-acid-CoA ligase	6.2.1.3	Fatty acids	Fatty-acyl-CoA
	Acyl-CoA dehydrogenase	1.3.8.7	Fatty-acyl-CoA	2-Enoyl-CoA
	Enoyl-CoA hydratase	4.2.1.17	2-Enoyl-CoA	(R)-3-hydroxyacyl-CoA
	Polyhydroxyalkanoic acid synthase	2.3.1.-	(R)-3-hydroxyacyl-CoA	Polyhydroxybutyrate (PHB)

## Data Availability

The data presented in this study are available in article.
